# Target Fortification of Breast Milk: How Often Should Milk Analysis Be Done?

**DOI:** 10.3390/nu7042297

**Published:** 2015-04-01

**Authors:** Niels Rochow, Gerhard Fusch, Bianca Zapanta, Anaam Ali, Sandip Barui, Christoph Fusch

**Affiliations:** 1 Division of Neonatology, Department of Pediatrics, McMaster University, 1280 Main Street West, Room HSC-4 F5, Hamilton, ON L8S4K1, Canada; E-Mails: nielsrochow@gmail.com (N.R.); gefusch@mcmaster.ca (G.F.); zapantabianca@gmail.com (B.Z.); anaamali@gmail.com (A.A.); 2 Department of Mathematics and Statistics, McMaster University, 1280 Main Street West, Hamilton, On L8S4K1, Canada; E-Mail: baruis@math.mcmaster.ca

**Keywords:** fortifier, enteral feeding, nutrition, postnatal development, personalized medicine, postnatal growth restriction

## Abstract

Target fortification (TFO) reduces natural macronutrient variation in breast milk (BM). Daily BM analysis for TFO increases neonatal intensive care unit work load by 10–15 min/patient/day and may not be feasible in all nurseries. The variation of macronutrient intake when BM analysis is done for various schedules was studied. In an observational study, we analyzed 21 subsequent samples of native 24-h BM batches, which had been prepared for 10 healthy infants (gestational age 26.1 ± 1.3 weeks, birth weight: 890 ± 210 g). Levels of protein and fat (validated near-infrared milk analyzer), as well as lactose (UPLC-MS/MS) generated the database for modelling TFO to meet recommendations of European Society for Paediatric Gastroenterology Hepatology and Nutrition. Intake of macronutrients and energy were calculated for different schedules of BM measurements for TFO (*n* = 1/week; *n* = 2/week; *n* = 3/week; *n* = 5/week; *n* = 7/week) and compared to native and fixed dose fortified BM. Day-to-day variation of macronutrients (protein 20%, carbohydrate 13%, fat 17%, energy 10%) decreased as the frequency of milk analysis increased and was almost zero for protein and carbohydrate with daily measurements. Measurements two/week led to mean macronutrient intake within a range of ±5% of targeted levels. A reduced schedule for macronutrient measurement may increase the practical use of TFO. To what extent the day-to-day variation affects growth while mean intake is stable needs to be studied.

## 1. Introduction

Breast milk is recommended as the first choice of nutrition for very low birth weight (VLBW, birth weight <1500 g) infants [1,2]. However, its nutritional composition may need to be optimized in order to meet the high demand for protein and energy required by premature infants due to their faster growth rates (15–22 *vs.* 6–9 g/kg/day in term infants) [3,4]. Currently, breast milk composition is enhanced by adding commercially available fortifiers. At the recommended dosage, common products provide extra protein (1–1.1 g/dL), but the amount and composition of non-protein calories vary considerably by the manufacturer (from 0 to 1 g/dL for fat and 0.4 to 3.4 g/dL for carbohydrates) [5,6]. The fixed dosage approach to fortification was developed assuming an average content of macronutrients and energy in breast milk. As such, it does not take into account the significant inter- and intra-individual variation of breast milk composition [7,8,9,10,11,12,13]. Due to this large real-life variation, the exact macronutrient intake of VLBW infants fed fortified breast milk in clinical practice is unknown. According to available published evidence, fixed dose fortified breast milk does not meet the recommended intake in approximately 25%–40% of VLBW infants due to inadequate low breast milk content for protein and/or calories [7,10,14]. These findings might contribute to the observation that up to 58% of VLBW infants fed fortified breast milk still experience postnatal growth restriction [15].

The aim of target fortification of breast milk is to individually tailor macronutrient content based on regular analysis of breast milk. Hence, it aims to “standardize” the composition of breast milk and provides infants with a consistent and defined intake [7]. However, the evidence base on short- and long-term outcomes, including growth and neurodevelopment achieved with target fortification of breast milk, is weak. In a previous study, we were able to show that weight gain is closely related to ingested volume of target fortified breast milk (*r*^2^ = 0.68), but not of standard fortified breast milk (*r*^2^ = 0.02) [7]. Growth rates seemed to be independent of milk intake in infants with standard fortified breast milk. We speculated that this was an effect of the natural variation of breast milk on growth rates [7].

Implementing target fortification adds additional workload to the neonatal intensive care unit (NICU) team. The total time required to analyze breast milk composition is approximately 5 to 7 min per sample. Additionally, time is needed for the calculation of target fortification, documentation and printing of the fortification prescription, which amounts to 5 to 10 min per sample [7]. In a 50-bed NICU with typically 80% of the infants on target fortified breast milk, this would lead to 7 to 12 h/day of additional workload for daily measurements. Hence, a reduced frequency of macronutrient measurements would help to make target fortification more practically in clinical routine. It is therefore the aim of the present study to model the impact of various frequencies of breast milk analysis measurements on the effective macronutrient intake of VLBW infants.

## 2. Methods

### 2.1. Study Design and Study Population

This observational study was performed at the NICU (Level 3) at McMaster Children’s Hospital in Hamilton, Ontario, Canada. A set of 210 samples were collected from pooled 24-h breast milk batches, which had been prepared for ten healthy, fully breast fed preterm infants during a 21-day period (gestational age of 26.1 ± 1.3 weeks, birth weight of 890 ± 210 g, head circumference of 25.2 ± 3.9 cm, day of life 30 ± 7, maternal age 29 ± 7 years). Such batches were usually pooled from one to three arbitrary samples. Preferably, fresh native breast milk was used. In cases when no fresh breast milk was available, frozen native breast milk was used by the order it was pumped and by volume to best match the required fluid amount and minimize discard. The collection of breast milk samples was initiated five days after the infants had reached full enteral feeds defined as a volume of 150 mL/kg/day (day of life 30 ± 7). Details about the study protocol were previously published [7].

Informed written parental consent was obtained prior to inclusion of infants into the study. The study was approved by the Research Ethics Board of McMaster University (REB#12-109) and registered at ClinicalTrials.gov under identifier NCT01305642.

### 2.2. Milk Analysis

A sample of 2 mL was obtained from each breast milk batch for macronutrient analysis. One milliliter was subsequently used to analyze protein and fat using a near-infrared milk analyzer (SpectraStar, Unity Scientific, Brookfield, CT, USA). Results were adjusted according to our detailed validation process as previously published [13]. In this study, lactose measurements were performed with ultra-performance liquid chromatography-tandem mass spectrometry (UPLC-MS/MS) to overcome the inherent imprecision of the milk analyzer for lactose [13]. Therefore, the remaining 1 mL of breast milk was stored at −80 °C and thawed later to measure its lactose content with UPLC-MS/MS. Samples were analyzed as previously described [16,17] and pipetted with a Biomek^®^ FX^P^ robotic system (Beckman Coulter, Brea, California) to enhance accuracy. Prior to both methods, near-infrared and tandem mass spectroscopy, breast milk samples were homogenized for 15 seconds using a sonicator (VCX 130, Sonic & Materials Inc., Newtown, CT, USA). The stability of lactose content at −80 °C has been recently reported [13,18,19,20].

### 2.3. Definitions

Fixed dose fortification (FDF), which has been also named as standardized (“blind”) fortification, is the addition of fortifier to breast milk in order to increase the level of nutrients, electrolytes, vitamins and trace elements in a fixed dose (g fortifier/dL milk). The dose of the fortifier is not dependent on the individual composition of native breast milk [10].

Target fortification (TFO) is an extension of the concept of fixed dose fortification. Based on individual breast milk analysis, those macronutrients are identified that still will be deficient after applying fixed dose fortification. Along with fixed dose fortification, individual modular fortifier products (protein and/or carbohydrate and/or fat) are calculated and added to achieve the target composition [7].

### 2.4. Calculation of Amount of Modular Fortifier

The amount of modular components (protein and/or carbohydrate and/or fat) needed for target fortified breast milk was calculated as previously described [7]. This algorithm consisted of three steps. First, the amount of macronutrients (protein, carbohydrate, fat) was measured in native 24-h breast milk batches. Second, the amount of macronutrients achieved after fixed dose fortification was calculated by adding the macronutrient levels measured in the native 24-h breast milk batch and the increment from the addition of fixed dose fortification at the recommended dosage. For this step, values given by the fortifier manufacturer’s data sheet were used (Enfamil HMF^®^, Mead Johnson, Evansville, Indiana, USA): a recommended dosage of 2.84 g fortifier per dL of breast milk provides an increment of 1.1 g protein, 0.4 g carbohydrate and 1.0 g fat (all per dL). As a third and final step, the amount of modular products (protein, carbohydrate and fat) required to achieve target values was calculated.

The following commercially available products were used: Microlipid^®^ (Nestle Health Care Nutrition, Minneapolis, MN, USA), a safflower oil fat emulsion developed for enteral feedings (0.5 g fat/mL); Beneprotein^®^ (Nestle, Health Care Nutrition, Minneapolis, MN, USA), an instant whey protein powder (0.86 g protein/g); and Polycose^®^ (Abbott Nutrition, Columbus, OH, USA), a glucose polymer powder (0.94 g carbohydrate/g).

The target macronutrient concentration was defined as 3 g/dL for protein, 8.5 g/dL for carbohydrate and 4.3 g/dL for fat. This definition is based on guidelines of European Society for Paediatric Gastroenterology Hepatology and Nutrition (ESPGHAN) in order to achieve an intake of 4.5 g/kg/day of protein, 12.8 g/kg/day of carbohydrate and 6.5 g/kg/day of fat at an enteral volume of 150 mL/kg/day [1]. In the case that a macronutrient component after fixed dose fortification already met target amounts, only the deficient macronutrient components were target fortified.

### 2.5. Description of Different Scenarios for Weekly Measurement Frequencies

To assess the impact of different workload scenarios in the NICU, five weekly schedules of milk analysis were simulated: (1) only Monday (*n* = 1/7); (2) Monday and Thursday (*n* = 2/7); (3) Monday, Wednesday and Friday (*n* = 3/7); (4) all weekdays, but no weekends (*n* = 5/7); and (5) every day (*n* = 7/7). Fortification and achieved intake were calculated for the total of 21 days. Schedules 1–4 assume that macronutrient content will not be measured every day. For days without measurements, milk analysis data from the most recent preceding day was used as a substitute, taking the likelihood into account that the true macronutrient contents between these days were different. For example, Schedule 4 assumes that milk analysis is done only on weekdays. In order to calculate the amount of modular products for TFO on weekends, the measurement of the preceding Friday was used as a substitute.

For the different schedules, the resulting intake using modular fortification was calculated, and all available data from daily analysis and the average over a period of the entire 21 days were computed. Energy intake was calculated based on the physiological energy equivalents (Atwater factor) assuming that fat yields 9 kcal per gram and protein and carbohydrate both yield 4 kcal per gram [21].

To check whether the targeted levels for macronutrients had been achieved, a deviation within ±5% from the targeted level had been defined as acceptable.

### 2.6. Statistics

The modeling of different approaches for target fortification and calculation of the mean, standard deviation, median, interquartile range, percentage and frequencies were done using Microsoft Excel 2013 (Microsoft Corporation, Redmond, WA, USA). Data were tested for a normal distribution using the Kolmogorov—Smirnov test. Box plots were calculated using the R software package for statistical analysis Version 3.1.0 (4 October 2014) [22].

## 3. Results

Table 1 summarizes the macronutrient contents of *n* = 210 in 24-h breast milk batches, before and after fixed dose fortification, as well as after target fortification using different schedules for bedside analysis. In native breast milk, there was considerable intra- and inter-individual variation for all macronutrients. The ranges were 0.5 to 2.0 g/dL for protein, 3.0 to 10.6 g/dL for carbohydrate (lactose), 2.0 to 6.0 g/dL for fat and 48 to 89 kcal/dL for energy. Fixed dose fortification with Enfamil HMF^®^ increased macronutrient content by 1.1 g/dL for protein, 0.4 g/dL for carbohydrate, 1 g/dL for fat and 15 kcal/dL for energy. On average, the protein content of fixed dose fortified breast milk was 2.3 g/dL and carbohydrate was 7.7 g/dL and did not reach the defined target levels (3 g protein/dL and 8.5 g carbohydrate/dL, respectively). Native fat content on average exceeded the target level (4.7 *vs.* 4.3 g/dL).

**Figure 1:**
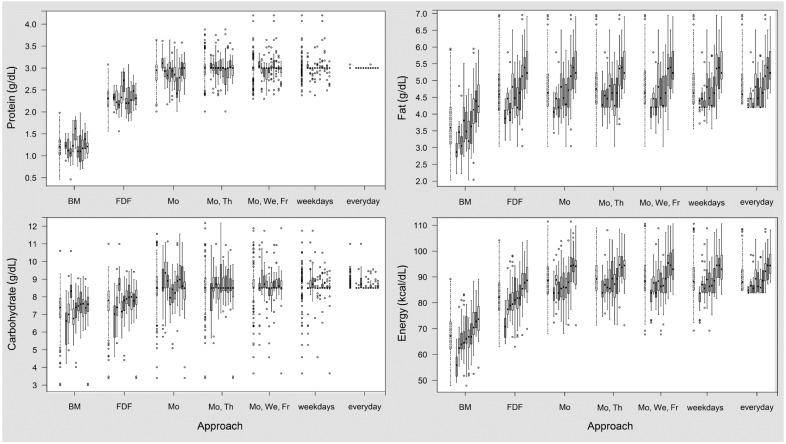
Variation of macronutrient and energy content in target fortified breast milk (BM) for different frequencies of measurements compared with native BM and fixed dose fortified (FDF) BM. The first boxplot of each group represents all samples (*n* = 210), followed by ten boxplots with data (*n* = 21) for individual subjects.

**Figure 2:**
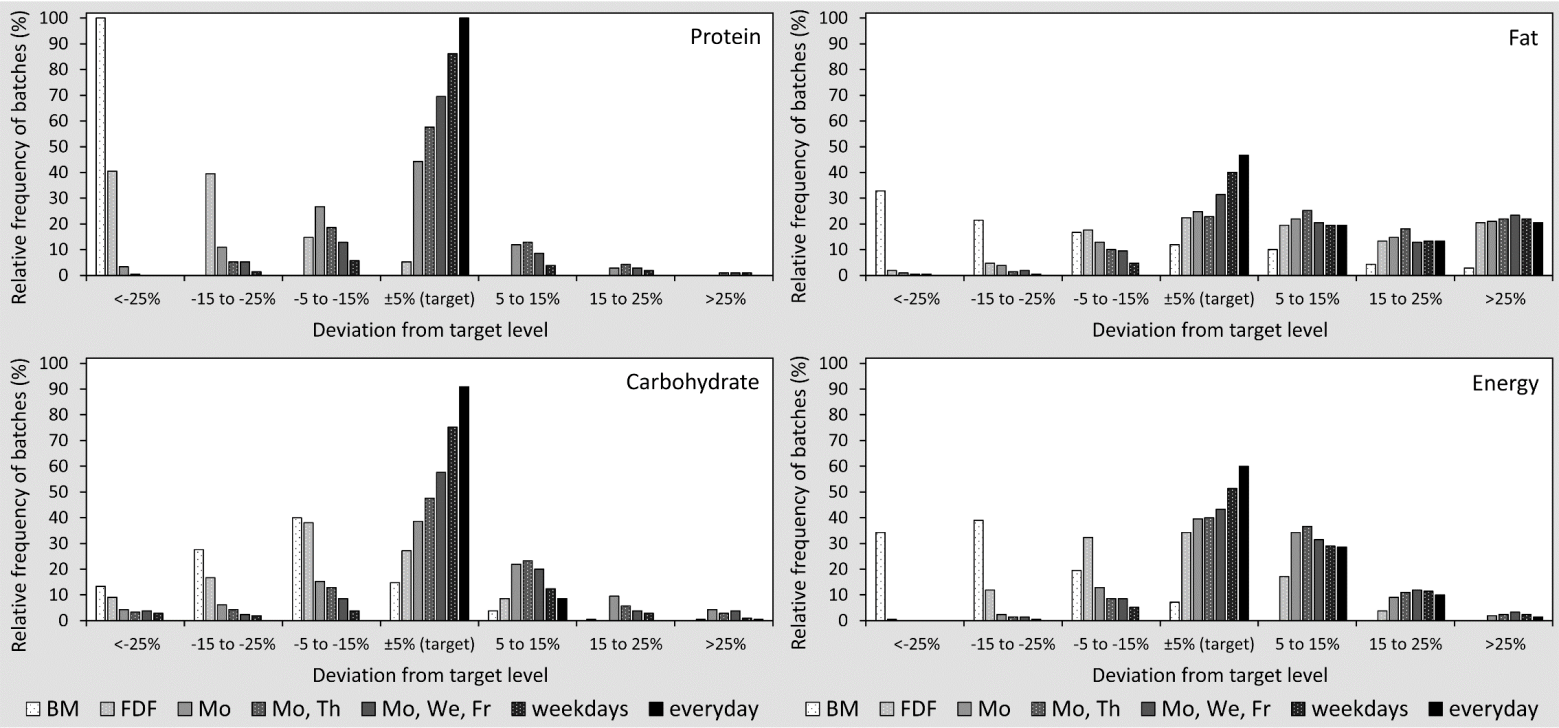
Percentage of samples with the deviation of macronutrients and energy content from ESPHGAN recommendations in single breast milk (BM) batches for native BM, fixed dose fortification (FDF) and target fortification (TFO) (five different schedules). The *X*-axis shows stratified ranges (deviation from target levels). The sum of relative frequencies per schedule is 100%.

In target fortified breast milk, daily measurement precisely met the target intake for protein and carbohydrates. The day-to-day variation of macronutrients increased as the frequency of milk analysis decreased. A detailed overview about the distribution of the differences of macronutrient amounts based on modelling of target fortification on different schedules is illustrated in Table 2 and in Figure 1 and Figure 2.

Looking closer into protein levels, a reduced number of measurements increased the variation considerably. For Schedules 2–5, mean protein intake during a 21-day period was within a range of ±5% of the target level. However, when measurement frequency was reduced to once per week (Schedule 1), 30% of the individuals would be at risk for a deviation of intake from the target level alongside a high day-to-day variation (Figure 1).

Similar to protein, the carbohydrate concentration achieved by target fortification attained precise target levels with daily measurements. Again, analysis twice a week seemed to be reasonably suited to supplement the average macronutrient content to the ESPGHAN target levels. Some milk batches showed a final carbohydrate content that was higher than targeted; however, this concentration was already achieved after adding the fixed fortifier and was not an effect of target fortification.

**Table 1:** Protein, carbohydrate, fat and energy of native breast milk (BM), fixed dose fortified BM and target fortified BM (TFO) for measurements of macronutrients done on different schedules.

Component	ESPGHAN Recommendation	Native BM	Amount Added by Fixed Dose Fortification	Fixed Dose Fortification	TFO	TFO	TFO	TFO	TFO
					Mo	Mo and Th	Mo, We, Fr	Weekdays	Daily
Protein (g/dL)	2.7–3.0 ^$^/2.3–2.7 ^#^	1.2 ± 0.3 *	1.1	2.3 ± 0.3 *	2.9 ± 0.3	3.0 ± 0.3	3.0 ± 0.2	3.0 ± 0.2	3.0 ± 0.0
		1.2 (1.0; 1.4)		2.3 (2.1; 2.5)	3.0 (2.7; 3.1)	3.0 (2.9; 3.1)	3.0 (2.9; 3.0)	3.0 (3.0; 3.0)	3.0 (3.0; 3.0)
Carbohydrate (g/dL)	7.7–8.8	7.3 ± 1.1	0.4	7.7 ± 1.1	8.6 ± 1.2	8.6 ± 1.1	8.6 ± 1.0	8.5 ± 0.8	8.6 ± 0.3
		7.4 (6.8; 7.9)		7.8 (7.2; 8.3)	8.5 (8.1; 9.1)	8.5 (8.3; 9.1)	8.5 (8.5; 9.0)	8.5 (8.5; 8.6)	8.5 (8.5; 8.5)
Fat (g/dL)	3.2–4.4	3.7 ± 0.8 *	1.0	4.7 ± 0.8 *	4.8 ± 0.8 *	4.8 ± 0.7 *	4.8 ± 0.7	4.8 ± 0.7	4.8 ± 0.6
		3.6 (3.1; 4.2)		4.6 (4.1; 5.2)	4.6 (4.2; 5.2)	4.8 (4.3; 5.2)	4.6 (4.3; 5.3)	4.6 (4.3; 5.2)	4.6 (4.3; 5.2)
Energy (kcal/dL)	73–90	67 ± 9 *	15	82 ± 9 *	89 ± 8 *	90 ± 7 *	90 ± 8	90 ± 7	90 ± 6
		67 (62; 73)		82 (77; 88)	89 (83; 94)	89 (85; 95)	88 (85; 95)	88 (85; 94)	88 (85; 93)

Data show the mean ± standard deviation, median (interquartile range). Normally distributed values are indicated by *. Though native BM contains only lactose, levels are labelled as carbohydrates, because fortifiers contain carbohydrates other than lactose. European Society for Paediatric Gastroenterology Hepatology and Nutrition (ESPGHAN) recommendation have been adjusted for a milk volume intake of 150 mL/kg/day. Protein intake for infants with body weight <1 kg is indicated by ^$^ and 1–1.8 kg by ^#^. Mo, Monday; We; Wednesday; Th, Thursday; Fr, Friday.

**Table 2:** Number of subjects affected by the deviation of mean macronutrients and energy intake from ESPHGAN recommendations. Data (*n* = 210 measurements of *n* = 10 subjects) presented for native breast milk (BM), fixed dose fortified BM and of target fortified (TFO) BM for measurements done on different schedules for BM analysis.

Component	Deviation from Target Composition (%)	Native BM	Fixed Dose Fortification	TFO	TFO	TFO	TFO	TFO
				Mo	Mo and Th	Mo, We, Fr	Weekdays	Daily
Protein	>25							
	15 to 25							
	5 to 15						1	
	±5 (target)			7	10	10	9	10
	−5 to −15		1	3				
	−15 to −25		4					
	<−25	10	5					
Carbohydrate	>25							
	15 to 25							
	5 to 15			4	1	2		
	±5 (target)		1	3	9	8	9	10
	−5 to −15		7	3			1	
	−15 to −25		2					
	<−25	10						
Fat	>25		1	1	1	1	1	1
	15 to 25		2	2	2	2	2	2
	5 to 15		3	3	3	4	4	4
	±5 (target)	3	3	3	4	3	3	3
	−5 to −15	2	1	1				
	−15 to −25	3						
	<−25	2						
Energy	>25							
	15 to 25							
	5 to 15			3	4	5	5	4
	±5 (target)		6	7	6	5	5	6
	−5 to −15	2	3					
	−15 to −25	6	1					
	<−25	2						

The routine fortifier for fixed dose fortification was Enfamil HMF^®^. Numbers of samples have been summarized according to the deviation from target macronutrient levels (protein 3 g/dL, carbohydrate 8.5 g/dL, fat 4.3 g/dL, energy 85 kcal/dL). Each block of data shows the distribution of the numbers of infants that would have mean or be cumulative in strata. TFO, target fortification; Mo, Monday; We; Wednesday; Th, Thursday; Fr, Friday.

In contrast to the modeling observations with protein and carbohydrates, the levels of fat in native breast milk had a higher variation, and 20% of native breast milk samples already exceeded the target levels for fat prior to any kind of fortification. After fixed dose fortification, the fat content was above the 5% tolerance range of the target level in 53% of breast milk samples. Target fortification decreased the number of fat-deficient samples, while the number of samples with fat amounts above target levels due to fixed dose fortification remained unchanged. Measurements twice per week eliminated the number of samples with low fat levels.

The energy content of breast milk showed a similar pattern compared to single macronutrients. The day-to-day variation decreased as the frequency of milk analysis increased.

## 4. Discussion

In this study, we investigated the variation of fat, protein, carbohydrate and energy of pooled native breast milk, fixed dose fortified breast milk and target fortified breast milk when fortification was based on different schedules of macronutrient measurements. The main findings of the study were: (1) fixed dose fortification of breast milk was not sufficient to provide macronutrients at the recommended level in all preterm infants; (2) target fortification with daily measurements precisely achieved recommended target intake; (3) the reduction of measurement frequency increased the day-to-day variation; (4) measurements twice a week assured that, on average, no infant received an intake that was below the recommended targets; and (5) the higher amount of carbohydrates and fat in some of the batches was due to the composition of native breast and/or fixed dose fortifier, but not an effect of target fortification.

In this study, we used daily samples of native 24-h breast milk batches collected during a 21-day period. The 24-h batches had been prepared in the same way as all other routine fortified breast milk feedings at our NICU. Usually, one to three portions of stored mother’s own milk were used for pooling. The breast milk analysis showed that the variation of protein, lactose and fat content that we observed in our study was in the same order of magnitude as previously reported for samples obtained from single lactations [8,23,24]. We are confident that our breast milk batches reflect real-life routine conditions, as we used a validated near-infrared milk analyzer for protein and fat, a validated wet chemistry method for lactose and excluded freezing and storage time as a confounder [13,16,17].

Recently, we demonstrated that growth rates correlated with milk volume intake when infants receiving breast milk with insufficient macronutrient content were identified and received target fortified breast milk instead. In infants receiving fixed dose fortification only, growth rates did not correlate with milk volume [7]. In that recent study, target fortification was based on daily measurements. However, if breast milk is not measured daily, day-to-day variation of macronutrient intake would rise, thereby increasing the probability that mean intake will deviate from the target value. In our study, target fortification ensured that the average intake of macronutrients reached the target levels when breast milk was analyzed at least twice a week. Currently, it is unknown whether the degree of day-to-day variation at constant average intake has an impact on metabolic response and growth in preterm infants, as has been shown in adults [25,26,27]. A reduced number of meals, but not the total caloric intake, led to higher fat mass accumulation [25]. Other studies kept the caloric intake constant, but varied protein and carbohydrate levels and found effects on glycemic response [26,27]. It might be speculated that some variation is physiological for mature babies, as in toddlers, it was found that intake of breast milk volume had an average day-to-day variation of 9% ± 5% [28].

Compared to term infants, the metabolism of preterm infants is immature, but supports considerably higher growth rates. Recent research confirms that the fetus is exposed *in utero* to a more constant supply and influx of nutrients [29,30]. Considering these data, it could be speculated that a continuous supply of nutrients might be desirable to support optimum growth of preterm infants. In contrast, the different schedules of TFO led to different magnitudes of day-to-day variations. It is of great interest to know whether an increased uneven influx affects growth, neurodevelopment and body composition of very premature infants when compared to a more even influx of calories and macronutrients. We feel that this is a valuable question, particularly in light of the DOHaD (developmental origin of health and disease) hypothesis and needs further consideration. A randomized controlled trial would be most appropriate to test if an even influx of nutrients is superior to a regime with a higher variation.

The analysis of fixed dose fortified breast milk showed that 76 out of 210 batches required extra fortification with fat, but 53% of samples had fat amounts that were higher than the desired target level. This could be explained by two reasons. First, the fat content of native breast milk is subject to high variation [13,23]. A considerable number of samples of native breast milk samples already reaches the target fat content. Secondly, the fat component of the fortifier used in this study is composed such that the recommended fixed dose fortification to enhance protein content by 1.1 g/dL will also increase fat content by 1 g/dL. It is of importance to note that the high final concentration of fat was already achieved by routine fixed dose fortification and was not an effect of target fortification.

One rationale to perform this study was to identify whether the workload associated with target fortification could be reduced. We feel that two measurements per week provide a reasonable balance between workload and improving nutrient intake. We further optimized the workload by placing the homogenizer for milk sample pretreatment and the infrared milk analyzer directly in the milk preparation room located in the NICU. This infrared milk analyzer works quickly and is simple to use, comparable to a blood gas machine. Dietician assistants had been trained to analyze samples drawn from pooled 24-h batches. The computation and printing of the prescription for the amount of fat, protein or carbohydrate needed for target fortification were automated using a predefined Excel sheet. The modular protein and carbohydrate products for target fortification were in powder form. To avoid the need for a scale, these products had been prepared in small packages of 0.2 g, 0.5 g, 1 g, 2 g and 5 g increments. Fat was provided as a liquid emulsion, which was drawn with a syringe. As a result, the additional workload on days with breast milk measurements to fortify a 24-h batch was less than 10 min [7] compared to 1–2 min on days without measurements. Hence, target fortification that measures breast milk content twice a week will lead to 30 min of extra work per week and per infant, which accumulates to 2–4 h for a 4–8-week intervention [7]. In return, improved somatic growth and development reduce the complications of neonatal care, shorten the length of stay and reduce costs [31,32,33,34].

## 5. Conclusions

The concept of target fortification may enable identifying breast milk batches with low nutrient content and, therefore, could decrease the risk of inappropriate growth. Implementing target fortification could be an option to overcome the uncertainty and variability of the macronutrient content of breast milk. In regards to the results of this study, we think that target fortification with measurements at least twice per week would offer an acceptable cost-benefit compromise that would be able to enhance macronutrient intake in line with current recommendations. Future clinical studies are needed to examine whether the magnitude of day-to-day variation of macronutrients due to different frequencies of measurement affects growth, metabolism and neurodevelopment of preterm infants.
